# Intra‐amniotic mesenchymal stem cell therapy improves the amniotic fluid microenvironment in rat spina bifida aperta fetuses

**DOI:** 10.1111/cpr.13354

**Published:** 2022-10-20

**Authors:** Xiaowei Wei, Wei Ma, Hui Gu, Dan Liu, Wenting Luo, Songying Cao, Shanshan Jia, Tianchu Huang, Yiwen He, Yuzuo Bai, Weilin Wang, Zhengwei Yuan

**Affiliations:** ^1^ Key Laboratory of Health Ministry for Congenital Malformation, Department of Pediatric Surgery, Shengjing Hospital China Medical University Shenyang People's Republic of China; ^2^ Department of Pediatric Surgery, Shengjing Hospital China Medical University Shenyang People's Republic of China

## Abstract

**Objectives:**

Spina bifida aperta (SBA) is one of the most common neural tube defects. Neural injury in SBA occurs in two stages involving failed neural tube closure and progressive degeneration through contact with the amniotic fluid. We previously suggested that intra‐amniotic bone marrow‐derived mesenchymal stem cell (BMSC) therapy for fetal rat SBA could achieve beneficial functional recovery through lesion‐specific differentiation. The aim of this study is to examine whether the amniotic fluid microenvironment can be improved by intra‐amniotic BMSC transplantation.

**Methods:**

The intra‐amniotic BMSC injection was performed using in vivo rat fetal SBA models. The various cytokine expressions in rat amniotic fluid were screened by protein microassays. Intervention experiments were used to study the function of differentially expressed cytokines.

**Results:**

A total of 32 cytokines showed significant upregulated expression in the BMSC‐injected amniotic fluid. We focused on Activin A, NGF, BDNF, CNTF, and CXCR4. Intervention experiments showed that the upregulated Activin A, NGF, BDNF, and CNTF could inhibit apoptosis and promote synaptic development in fetal spinal cords. Inhibiting the activity of these factors weakened the anti‐apoptotic and pro‐differentiation effects of transplanted BMSCs. Inhibition of CXCR4 activity reduced the engraftment rate of BMSCs in SBA fetuses.

**Conclusion:**

BMSC transplantation can improve the amniotic fluid environment, and this is beneficial for SBA repair.

## Graphical abstract

In utero intra‐amniotic BMSC or PBS microinjection in the E15 fetuses was performed in E15 rat fetuses with spina bifida aperta, and amniotic fluid was collected at E21 for protein array detection. Venn diagram shows the relationship of three biological processes (GO: 0030335, 0048699, and 0043524) and the attribution of differentially expressed proteins. Comparative analysis of five proteins with the largest fold changes in the process of generation of neurons.

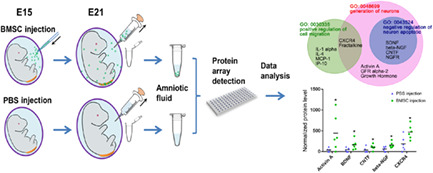

## 1 INTRODUCTION

Neural tube defects (NTDs) are congenital malformations of the nervous system, affecting about 300,000–400,000 infants worldwide.^1^ Spina bifida aperta (SBA) and anencephaly are two of the most common NTDs, resulting from the incomplete closure of the posterior and anterior neural tubes, respectively.^2^ Children with spina bifida have a high probability of lifelong physical and mental handicaps, and anencephaly is fatal. Remarkable advances in the fetal diagnosis of SBA and the availability of fetal therapies have allowed for intrauterine intervention to treat spina bifida, resulting in improved neurological function and increased life expectancy. However, the existing treatments cannot completely eliminate the serious disability or premature death of SBA individuals caused by nerve injury. Regular monitoring, continuous therapy, and medical and/or surgical treatments are often required to prevent and treat complications throughout the patient's life. The lifetime cost of caring for a child born with SBA is estimated at more than US$600,000.^3^, ^4^ Based on the two‐hit theory, neural injury in SBA occurs in two stages involving primary and secondary neural injuries. Disability in SBA results not only from failed neural tube closure but also from its progressive neurodegeneration through contact with the amniotic fluid.^5^ The primary neurulation failure is directly associated with failed neural tube closure, while the subsequent neurological damage might be reduced by covering the open neural tube through fetal surgical repair. Prenatal surgical repair has been performed in many centers in both Europe and the United States.^6^, ^7^, ^8^, ^9^, ^10^, ^11^, ^12^, ^13^, ^14^, ^15^ The Management of Myelomeningocele Study (MOMS) showed that fetal surgery closure between 19 and 26 weeks of gestation significantly offered the child decreased hydrocephalus shunting and ventriculo‐peritoneal placement rates, and also improved lower extremity function and bladder dysfunction.^16^, ^17^, ^18^, ^19^ Nevertheless, MOMS also reported the increased risks of intraoperative complications, preterm rupture, and uterine dehiscence after fetal surgery.^16^, ^20^ Even recently, the development of fetal endoscopy and mini hysterotomy showed a reduced maternal morbidity; however, the iatrogenic prematurity and neurological impairment are still a major complication of intrauterine surgery for SBA.^21^, ^22^, ^23^, ^24^ All of these prenatal repairs can only be performed in the second trimester of pregnancy, at which point the neural damage caused by SBA may be irreversible. In addition, fetal surgery consists of only defect closure and cannot treat primary nerve injury. Most SBA individuals who undergo prenatal repair continue to suffer from life‐long disabilities, such as sensory and motor weakness in the leg and fecal or urinary incontinence, even after fetal treatment. For these reasons, exploring an effective method for the early prenatal treatment of SBA before irreversible neural injury occurs is an important goal.

Bone marrow–derived mesenchymal stem cells (BMSCs), with a high capacity for self‐renewal; easily availability; the potential to differentiate into tissue‐specific cell types such as neurons, skin cells, skeletal muscle cells, and bone; and a low immunogenicity that permits allogenic transplantation without immunosuppressive drugs, have become a promising treatment for neural diseases.^25^, ^26^, ^27^ In animal models with spinal cord injuries (SCIs), transplanted MSCs and MSC‐derived microvesicles resulted in a better pro‐regenerative environment, which promoted neural stem cell survival, differentiation, and outgrowth of corticospinal axons.^28^ Transplanting MSCs into a rat model of traumatic brain injury can enhance endogenous cellular proliferation at the area of the brain via expressing neuro‐regulatory molecules.^29^ In vitro experiments indicated MSCs can promote neuron survival and neurite outgrowth through expressing neurotrophins and neuro‐regulatory proteins.^30^ We have previously successfully established intrauterine BMSC transplantation techniques for treating NTDs and have shown that transplanted BMSCs repaired multiple tissue defects and improved neural function recovery through multi‐differentiation and reducing neuronal apoptosis in in vivo and ex vivo rat NTD models.^31^, ^32^, ^33^, ^34^, ^35^ Besides the ability to replace diseased or damaged neurons via cellular differentiation, the paracrine effects of BMSCs may also participate in the treatment of NTD after BMSC transplantation. It has been reported that the amniotic fluid environment is toxic for cells in the exposed neural tube, and that this is the main cause of neural secondary injury.^36^, ^37^ This suggests that improving the amniotic fluid microenvironment may enhance the therapeutic effect on neural injury of NTDs, but whether intra‐amniotic BMSC transplantation can alleviate the neural injury by improving the amniotic fluid microenvironment has not been reported.

In this study, we for the first time explored whether the amniotic fluid environment that leads to secondary damage can be improved by intra‐amniotic BMSC transplantation within ex vivo and in vivo rat fetal NTD models. Through protein microassays and intervention experiments, we found and verified the factors involved in the intra‐amniotic injected BMSC‐induced NTD recovery. The method of BMSC delivery into the amniotic cavity is less traumatic for both mothers and fetuses and could provide a feasible approach to correcting malformations in early embryos. This approach can not only treat primary neural injuries but also solve the bottleneck problem in the narrow time window for prenatal surgery during which the neural damage caused by SBA may be irreversible. It can be applied alone or as an adjuvant to surgical treatment for NTD in the future.

## 2 MATERIALS AND METHODS

### 2.1 Experimental animals

Outbred Wistar rats (10–12 weeks old, 220–280 g; 4 weeks old, 80–100 g) were purchased from the Animal Center of China Medical University. All rats were kept under specific‐pathogen‐free (SPF) conditions with a 12 h light/dark cycle. Food and water were always supplied ad libitum during the experiments. The rats were mated overnight. The morning on which sperm cells were observed in the vaginal smear was determined as embryonic day 0 (E0). All procedures adhered to the National Institute of Health Guide for the Care and Use of Laboratory Animals and were approved by the Committee for Animal Care at China Medical University.

### 2.2 Isolation, culture expansion, and transfection of BMSCs

The BMSCs were isolated from the bone marrow of four‐week‐old Wistar rats and expanded and identified as previously reported.^31^ Briefly, the Wistar rats were euthanized with pentobarbitone sodium (40 mg/kg body weight). Then, the femoral bone marrow was flushed with DMEM/F12 (HyClone, USA) and centrifuged at 800 rpm for 5 min. The cell pellets were resuspended and cultured in DMEM/F12 supplemented with 10% fetal bovine serum (FBS) (10099141C; Gibco, USA), 100 IU/ml penicillin, and 100 μg/ml streptomycin (Gibco) in 25 cm^2^ tissue culture flasks (BD Biosciences, USA). The primary isolated BMSCs were defined as P0. The cultured BMSCs expressed CD90 (551401; BD Biosciencess) and CD29 (561,796; BD Biosciences), but not CD34 (sc7324; Santa Cruz Biotechnology, USA) and CD45 (sc1187; Santa Cruz Biotechnology), as revealed by flow cytometry using specific antibodies following previously published methods.^31^ At confluency, the cells were passaged (1:2) with fresh medium, and the P3 or P4 cells were used for the intra‐amniotic injection. The EGFP‐expressing adeno‐5 vector (100 pfu/cell; Hanbio, Shanghai, China) was transfected into the cultured cells for 24 h before transplantation to visualize the transplanted BMSCs. The transfected BMSCs were trypsinized, centrifuged (800 rpm), and resuspended in phosphate buffered saline (PBS; BI, Israel).

### 2.3 In utero intra‐amniotic BMSC injection

For the fetuses in utero, SBA was induced with a single intra‐gastric gavage of all‐trans retinoic acid (atRA; Sigma–Aldrich; 4% wt/vol in olive oil; 140 mg/kg body weight) to pregnant rats on E10 as previously described.^31^, ^38^

In utero intra‐amniotic BMSC microinjection was performed on E15 embryos. Pregnant rats were anesthetized with pentobarbitone sodium (40 mg/kg body weight). An incision was made in the abdominal wall, and the uterus was exteriorized. Under the operation microscope, the skin defect diameter of SBA fetuses was measured with an eyepiece micrometer. The fetuses with lumbo‐sacral spina bifida and skin defect diameter of 1.5–2 mm were chosen and randomly divided into the BMSC‐ (*n* = 25 fetuses) and PBS‐injected (*n* = 22 fetuses) groups. Using a micropipette, a suspension of GFP + ve BMSCs (2 μl, 5 × 10^6^ cells) or PBS was injected into the amniotic fluid via the uterine wall by the ventral aspect of the fetus, with care to avoid the fetus, the placenta, and the umbilical cord, and the locations of the injected fetuses were recorded. For the neurotrophic factor or their neutralizing antibody treatment, the fetuses were intra‐amniotically injected with 5 μg of human Activin A (AF‐120‐14; PeproTech, USA), human beta‐nerve growth factor (hNGF) (AF‐450‐01; PeproTech, USA), rat brain‐derived neurotrophic factor (rBDNF) (AF‐450‐02; PeproTech, USA), rat ciliary neurotrophic factor (rCNTF) (P20294.1; GenScript, China), anti‐Activin A (PA5‐47004; Invitrogen, USA), anti‐NGF (PA1‐24828; Invitrogen, USA), anti‐BDNF (PA1‐18363; Invitrogen, USA), anti‐CNTF (MA5‐23730; Invitrogen, USA), and equally mixed anti‐Activin A, anti‐BDNF, anti‐CNTF, and anti‐NGF suspended in 2 μl of PBS or the BMSC suspension (5 × 10^6^ cells) (*n* = 8 fetuses/group). After injections, the uterus was returned to the abdomen, and the abdominal wall was closed. The pregnant rats recovered from the anesthesia within 1 h and were returned to their home cage. The pregnant rats were euthanized at E21 by an overdose of pentobarbitone sodium, and the injected fetuses were extracted. The images of the fetuses were taken using a fluorescence stereomicroscope (M165FC, Leica, Germany) fitted with a Nikon DS‐Qi2 digital camera (NY‐1S35, Nikon).

### 2.4 Whole embryo culture (WEC), atRA treatment, and intra‐amniotic injection ex vivo

Pregnant females were euthanized at E10, and the embryos were dissected from the uterus and cultured as previously reported.^33^, ^39^ Briefly, embryos with intact yolk sacs and ectoplacental cones were placed in sealed 50 ml culture bottles (three embryos per bottle) containing 3 ml of sterile heat‐inactivated rat serum supplemented with 2 mg/ml of glucose. Culture bottles were placed in a roller apparatus and rotated at 25 rpm in a 37°C incubator with a continuous supplement of a gas mixture, including different concentrations of oxygen (5% O_2_ for the first 18 h, 20% O_2_ from 19 to 36 h, and 60% O_2_ from 37 to 48 h), 5% CO_2_, and balanced with N_2_.

To induce NTDs in the cultured embryos, atRA was added to the culture medium at final concentrations of 3 μM.^33^ Control cultures contained 0.1% DMSO (v/v). After 12 h, the embryos were transferred to fresh medium and cultured for another 36 h. For the ex vivo intra‐amniotic injection, C–X–C motif chemokine receptor (CXCR4) inhibitors (AMD3100; Sigma, USA) suspended in 0.2 μl of PBS or GFP + ve BMSC suspension (500 cells) were injected into the amniotic cavity of the embryos with a glass micropipette connected to a Hamilton syringe (*n* = 6 embryos/group). Approximately 500 cells (suspended in 0.2 μl of PBS) were injected per embryo. The micropipettes for the injection were made from borosilicate glass capillaries (model GD‐1; Narishige Scientific Instruments) on a micropipette puller (model PB‐7; Narishige Scientific Instruments). After culture, the amniotic cavity was incised, and the engraftment and distribution of GFP + ve BMSCs in the embryos were examined. Images were taken with a DS‐Qi2 CCD camera (NY‐1S35, Nikon).

### 2.5 Protein microassay of the amniotic fluid

The amniotic fluid with or without BMSC injection was drawn from the E21 SBA fetuses (*n* = 6 amniotic fluid samples/group). The RayBio® Biotin Label‐based Rat Antibody Array 1 (AAR‐BLG‐1; RayBiotech, USA) was used to determine the levels of 90 cytokines in the amniotic fluid according to the manufacturer's instructions. The fluorescence signal intensities of each protein were obtained using an InnoScan 300 Microarray Scanner (Innopsys). Background removal was performed, and the images were quantified using densitometry. A protein that had a weak normalized value of less than 10 was considered as not expressed in the amniotic fluid. By comparing the signal optical densities, the relative expression levels of cytokines were obtained. Positive controls were used to normalize the results from the different groups being compared. The fold‐changes in the protein expression were calculated by dividing the average optical density of the BMSC injection group by that of the PBS injection group. We used “fold change > 1.5 and probability values (*p*) < 0.05” as the threshold to judge the significance of protein expression difference. To generate the heatmap, we collected the optical density values of the differentially expressed proteins (fold‐change > 1.5 and *p* < 0.05) in all the samples and generated a matrix with the samples as rows and the proteins as columns. We chose raw as the log transform function to convert the optical density value then scaled the data by row for visualization using the ImageGP platform (http://www.ehbio.com/Cloud_Platform/front/). To gain insight into the biological changes in the amniotic fluid of the BMSC‐injected fetuses compared with the PBS‐injected fetuses, the differentially expressed proteins were categorized according to the Gene Ontology (GO) class “biological process.”

### 2.6 Tissue preparation and immunofluorescence

The embryos treated ex vivo were directly fixed in freshly prepared 4% paraformaldehyde (Sinopharm Chemical Reagent Co., Ltd, Shanghai, China) at 4°C for 24 h. The fetuses treated in vivo were perfused transcardially at E21 with 15 ml of physiologic saline, followed by 25 ml of 4% paraformaldehyde. After that, the lumbosacral spinal column containing muscle, spinal cord, and subcutaneous tissue was dissected and post‐fixed in 4% paraformaldehyde at 4°C for 24 h. The embryos or spinal columns were then cryoprotected in 20% sucrose for 24 h, embedded in Optimal Cutting Temperature compound, and sectioned into 30 μm serial sections using a freezing microtome (Microm hm525; Thermo, Germany). GFP + ve cells were observed by fluorescence microscopy (80i, Nikon). Sections with GFP + ve cells were marked and kept at −80°C in the dark for further immunofluorescence analysis. For the cultured cell staining, the BMSCs were seeded in 35‐mm glass bottom dishes (In Vitro Scientific) and fixed with 4% paraformaldehyde for 30 min, then perform immunofluorescence staining.

The primary antibodies used for the immunofluorescence were Nestin (MAB353; Millipore, USA), glial fibrillary acidic protein (GFAP), (MAB3402; Millipore, USA), β‐III Tubulin (TUJ1) (5568 S; Cell Signalling, USA), Activin A (PA5‐47004; Invitrogen, USA), GFP (AG279, AG281; Beyotime Institute of Biotechnology, China), BDNF (NBP2‐42215; Novus Biologicals, USA), CNTF (MA5‐23730; Invitrogen, USA), NGF (AB1528SP; Millipore, USA), and CXCR4 (ab124824; Abcam, USA). The secondary antibodies included Alexa Fluor 488‐conjugated goat anti‐rabbit (O‐11038; Invitrogen, USA) or anti‐mouse IgG antibody (A11001; Invitrogen, USA) and Rhodamine‐conjugated goat anti‐mouse (AP124R; Millipore, USA) or anti‐rabbit IgG antibody (AP132R; Millipore, USA). The immunofluorescence analysis was performed according to standard procedures. Images were taken with a C1 confocal microscope (Nikon). To determine the percentage of transplanted BMSCs that expressed specific cell markers, all of the GFP‐positive cells that were also immunopositive for the indicated cell markers were counted in each section. The percentages of BMSCs expressing Nestin, GFAP or TUJ1 were reported as the number of double‐positive cells/total number of GFP + ve cells/field.

### 2.7 Terminal‐deoxynucleoitidyl transferase/(TdT‐)mediated nick end labeling (TUNEL) analysis and immunofluorescence

The TUNEL analysis and immunofluorescence for the GFP were performed using the In Situ Cell Death Detection Kit (11684817910; Roche, USA) and the mouse anti‐GFP antibody (Beyotime Institute of Biotechnology) according to the manufacturer's protocol. Briefly, the sections were permeated with proteinase K (P9460; Solarbio, China) and blocked with PBS containing 10% FBS and 0.1% Triton x‐100. Then, the sections were incubated with mouse anti‐GFP antibody (AG271; Beyotime Institute of Biotechnology, China) at 4°C overnight. After washing with PBS, the TUNEL reaction mixture and Rhodamine‐conjugated goat anti‐mouse antibody (AP124R; Millipore, USA) were added to the sections and incubated at 37°C for 1.5 h. After washing, the sections were stained with DAPI and mounted with anti‐fade mounting medium. The images were taken with a C1 confocal microscope (Nikon, Japan). The number of total TUNEL in the neural tube of each section was then counted.

### 2.8 Immunoblot analysis

The protein extract (50 μg) was separated using 12.5% SDS‐PAGE and then transferred with Tris–HCl methanol (20 mM Tris, 150 mM glycine, 20% methanol) onto polyvinylidene difluoride membranes (Millipore, USA) in a trans‐blot electrophoresis transfer cell (Bio‐Rad). The blots were probed with antibodies against BCL2 (B9804; Sigma, USA), BAX (14796; Cell Signaling, USA), SYT (MAB5200; Millipore, USA), SYN (5297S; Cell Signaling, USA), or GAPDH (60004‐1‐Ig; Proteintech, USA). All immunoblotting was performed a minimum of three times. Immunopositive bands were visualized using enhanced chemiluminescence (WBSH0500; Millipore, USA) and quantified by the ImageJ software. The relative density of each protein was calculated by dividing the optical density of the protein by that of the loading control (GAPDH).

### 2.9 Statistical analysis

All analyses were performed in a double‐blind manner. The data are presented as the mean ± SEM. For single comparisons, we used Student's unpaired *t*‐test. For comparisons of more than two groups, a one‐way analysis of variance (ANOVA) followed by the Bonferroni's post‐test was used. The statistical tests were two‐sided with a significance level of the *p*‐values <0.05. All data were analyzed using SPSS 21.0.

## 3 RESULTS

### 3.1 SBA‐lesion specific migration and differentiation of the intra‐amniotic injected BMSCs in the embryos/fetuses in vivo

**FIGURE 1:**
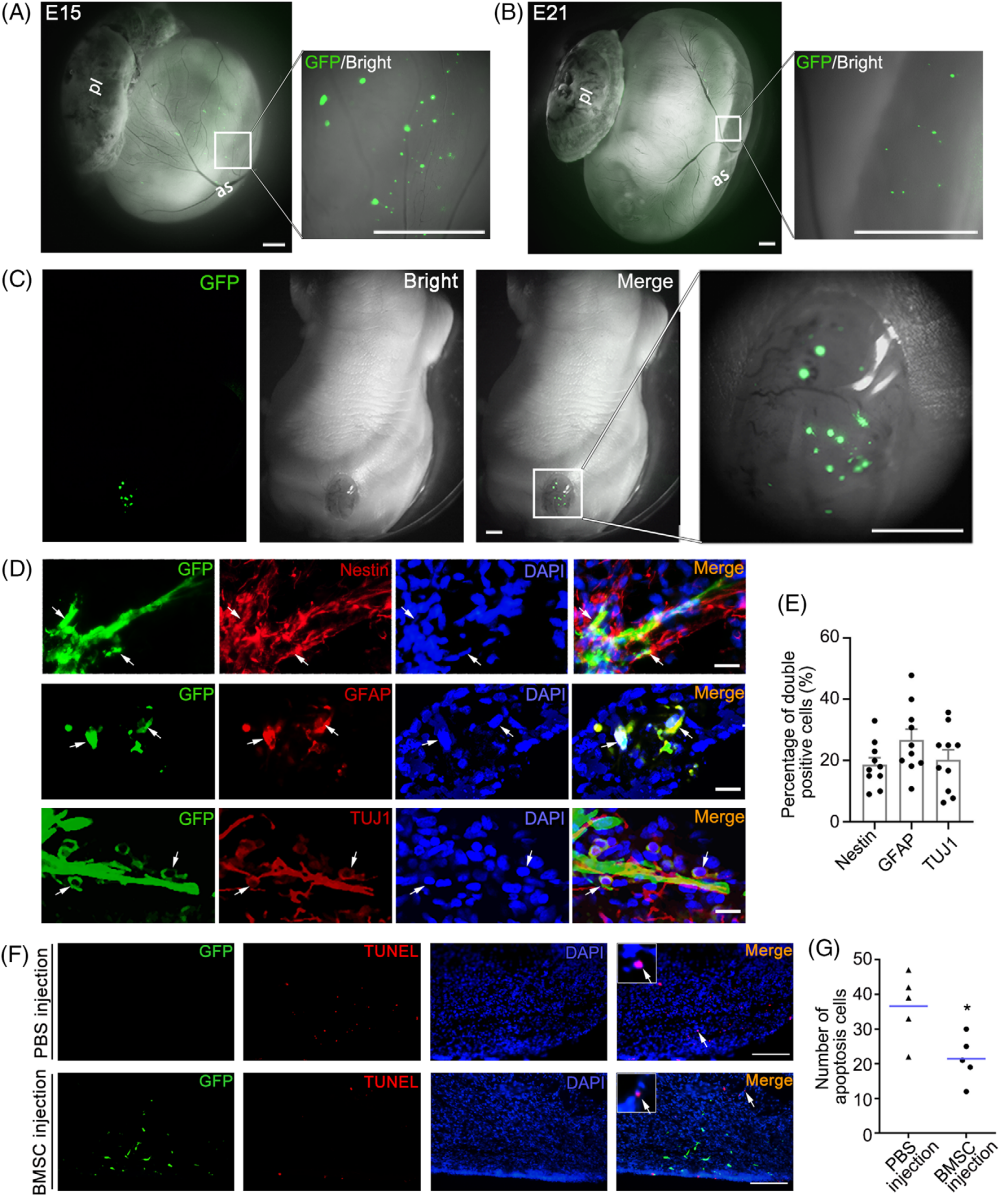
GFP‐labeled BMSCs specifically engraft into SBA‐lesions, differentiate into neural lineage cells, and inhibit cell apoptosis in defective spinal cords. (A) Representative fluorescent stereomicroscopic images of an E15 rat fetal sac that has just received intra‐amniotic BMSC transplantation from ventral side of the fetus, and a large number of scattered cells can be observed in amniotic fluid. Scale bars: 2.5 mm. Abbreviations: pl, placenta; as, amniotic sac. (B) Representative fluorescent stereomicroscopic images of an E21 rat fetal sac with intact amniotic membranes and many GFP + ve BMSCs suspended in the amniotic fluid. Scale bars: 2.5 mm. (C) Representative fluorescent stereomicroscopic images of a rat fetus with engrafted GFP + ve BMSCs (green) in the defect area after intra‐amniotic BMSC transplantation. The white box indicates the defective region of the SBA fetus that is enlarged on the right. Scale bars: 2.5 mm. (D) Representative immunofluorescence images showing engrafted BMSCs expressing Nestin (red, top row), GFAP (red, middle row), and TUJ1 (red, bottom row), which are dual‐labeled with GFP (green) in the tissue section of the defective neural tube after intra‐amniotic transplantation. The double positive cells are marked by arrows. Nuclei were stained blue with DAPI. Scale bars: 50 μm. (E) Quantitative analysis of Nestin, GFAP, and TUJ1 positive cells in defective spinal cords with BMSC engraftment. The percentage of BMSCs expressing these markers was determined as the number of double positive cells/total number of GFP positive cells (*n* = 10 sections/group). (F) Representative confocal microscopic images showing decreased apoptosis (red dots) in the spinal cord after intra‐amniotic BMSC (green dots) injection (below row) compared with PBS injection (upper row). The typical apoptotic cells indicated by the white arrows are magnified in the upper left corner of the images. Scale bars: 200 μm. (G) Quantification of the apoptotic cells was performed under a 40× field (*n* = 5 sections/group); the number of apoptotic cells in the dorsal spinal cord of the BMSC‐injected group was significantly decreased compared to that in the PBS‐injected group. *Significant difference compared with the PBS‐injected group, *p* < 0.05. Scale bar: 100 μm.

We performed an in utero intra‐amniotic BMSC injection in the E15 fetuses, then it could be observed that the injected BMSCs which were labeled by GFP evenly distributed in amniotic fluid (Figure 1A). Six days later, we observed the fetuses following the intra‐amniotic injections and found that there still were some GFP + ve BMSCs that persisted in the amniotic fluid and membrane in the E21 fetuses (Figure 1B), and a number of GFP + ve BMSCs specifically migrated into the defective spinal cords and survived in the SBA lesion of E21 fetuses (Figure 1C).

To investigate whether the engrafted BMSCs could generated different types of cells in the recipient spinal cords, we examined the expressions of specific neural lineage markers in the engrafted BMSCs using immunofluorescence double staining. The results showed that engrafted BMSCs in the defective spinal cords expressed markers of neural precursor cells (Nestin), neurogliocytes (GFAP) and neurons (TUJ1) 6 days after transplantation. The results showed that 18.6 ± 2.2% of the engrafted BMSCs expressed Nestin, 26.7 ± 3.4% expressed GFAP, and 20.2 ± 3.1% expressed TUJ1 (Figure 1D,E). The TUNEL analyses showed that positive apoptotic cells were frequently detected in the defect area of the spinal cord in fetuses injected with the PBS. In contrast, apoptotic cells were rarely found in the defective spinal tissue near the region of the BMSC engraftment (Figure 1F,G). In addition, apoptosis was not observed in the transplanted BMSCs.

### 3.2 Changes in the cytokine expression in the amniotic fluid after the BMSC transplantation

**FIGURE 2:**
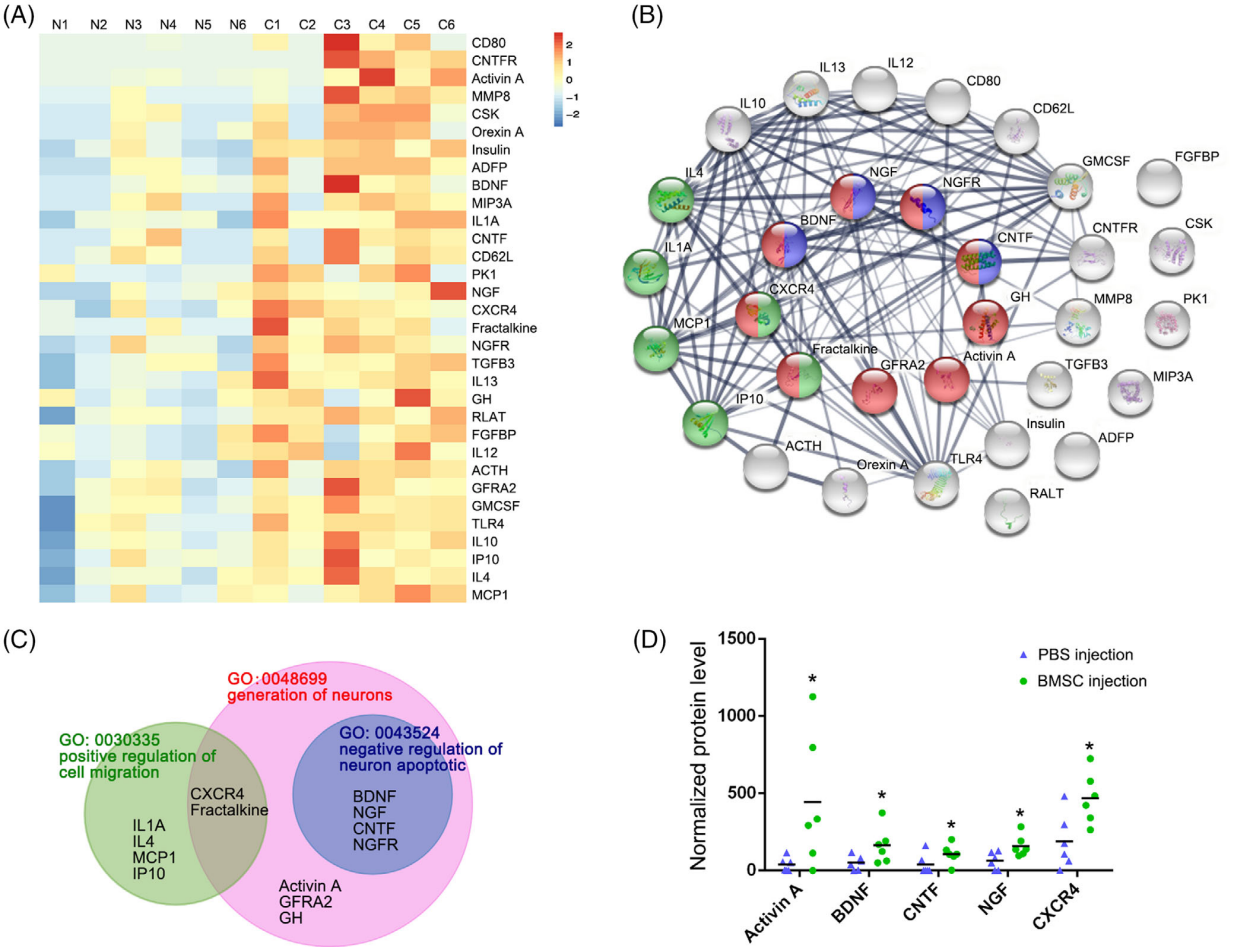
In utero intra‐amniotic BMSC injection altered the amniotic fluid microenvironment. (A) Heatmap showing the differentially expressed proteins in the amniotic fluid between the intra‐amniotic PBS injection (*n* = 6, sample: N1, N2, N3, N4, N5, N6) and BMSC injection (*n* = 6, sample: C1, C2, C3, C4, C5, C6) by a protein array analysis. Differentially expressed proteins (fold‐change ≥1.5 and *p* < 0.05) are arranged from high to low according to the fold changes. The fold changes were calculated by dividing the average optical density of the BMSC injection group by that of the PBS injection group. (B) Interaction network of differentially expressed proteins generated by the STRING database. Colors of the inside nodes indicate that the proteins come from different biological processes, including the process of generation of neurons (red), positive regulation of cell migration (green), and negative regulation of neuron apoptotic (blue). Proteins not in the three processes mentioned above are marked in gray. Lines connecting the nodes represent protein–protein associations, and the thickness of the line represents the edge confidence. (C) Venn diagram show the relationship of three biological processes (GO: 0030335, 0048699, and 0043524) and the attribution of differentially expressed proteins. (D) Comparative analysis of five proteins with the largest fold changes in the process of generation of neurons. *Significant difference compared with the PBS‐injected group, *p* < 0.05.

Considering that the paracrine effects of BMSCs, the survived BMSCs may affect the composition of amniotic fluid after entering amniotic fluid. The cytokines in the amniotic fluid play an important role in the specific migration and neural differentiation of BMSCs, and reduction of apoptosis in the spinal cord. To examine whether these BMSCs altered the cytokine composition of the amniotic fluid, we compared the expression of 90 cytokines in the amniotic fluid of BMSC‐ and PBS‐injected fetuses. A total of 32 cytokines showed significant upregulated expression between the two groups (Figure 2A). According to the biological process classifications, we focused on the differentially expressed cytokines involved in the SBA recovery. The protein expression of Activin A, BDNF, CNTF, NGF, NGFR, GFR, CNTF, growth hormone (GH), CXCR4, and Fractalkine in the generation of neurons; of NGF, BDNF, CNTF, NGFR in the negative regulation of neuron apoptotic process; and of CXCR4, Fractalkine, CCL2, IL1a, IL4, and IP10 in the positive regulation of cell migration all increased in the BMSC‐injected amniotic fluid. (Figure 2B,C). Among these differentially expressed proteins, Activin A, BDNF, NGF, CNTF, and CXCR4 are the five proteins with the largest fold changes, which means that the expression of these five proteins in the amniotic fluid upregulated most significantly after intra‐amniotic BMSC transplantation (Figure 2D).

### 3.3 Cytokine induction in the spinal cords due to BMSC transplantation

**FIGURE 3:**
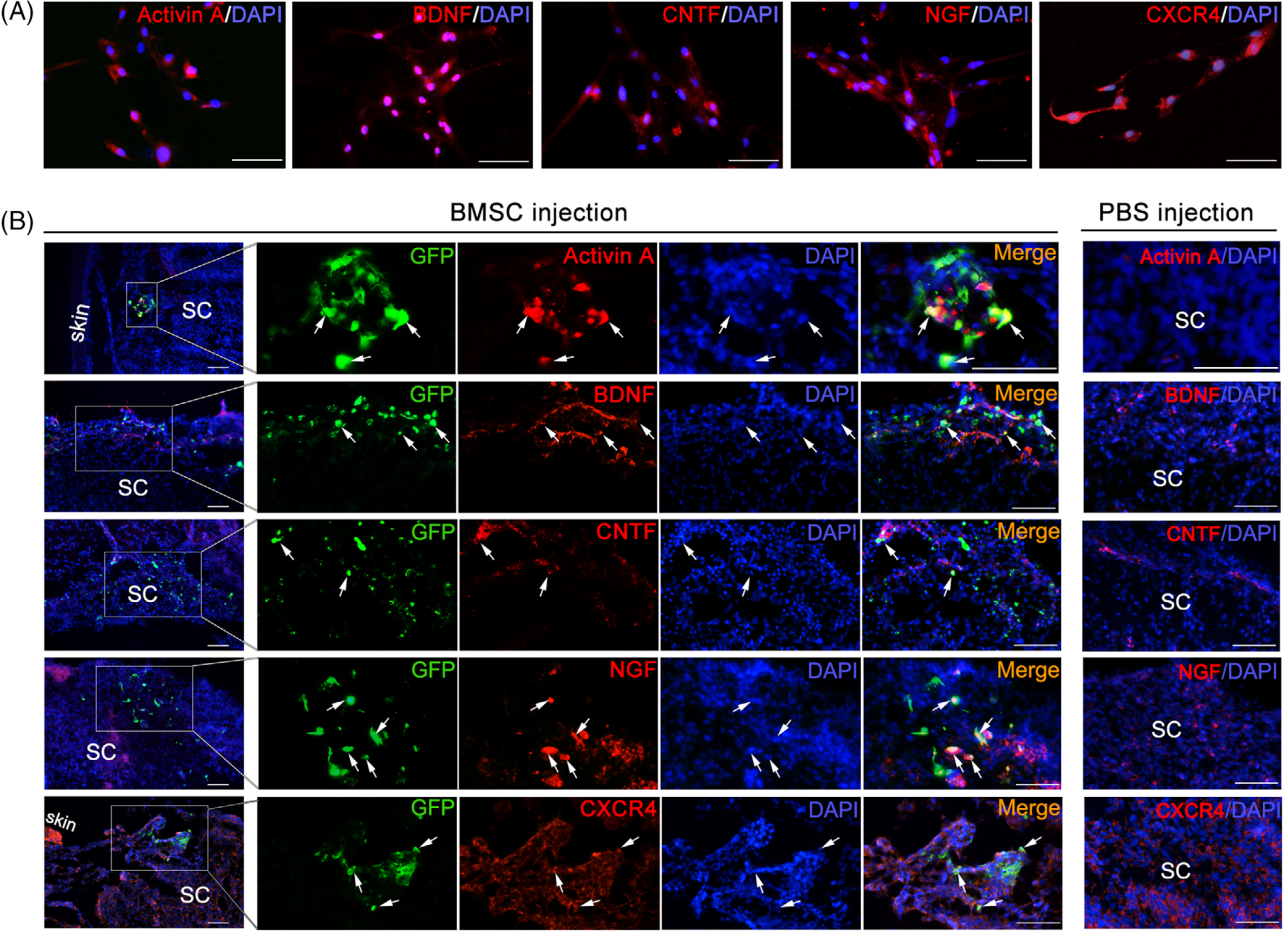
Engrafted BMSCs express Activin A, BDNF, CNTF, NGF, and CXCR4. (A) Immunofluorescence staining for Activin A, BDNF, CNTF, NGF, and CXCR4 (red) in the BMSCs cultured in vitro. Scale bar: 50 μm. (B) Immunofluorescence staining for Activin A, BDNF, CNTF, NGF, and CXCR4 (red) with GFP (green) in the sections of PBS‐injected and BMSC‐injected spinal cords. The nuclei stained by DAPI are blue. Typical triple positive cells are labeled with arrows. Scale bar: 50 μm.

To determine whether the increased expression of these five cytokines (Activin A, NGF, BDNF, CNTF, and CXCR4) in the amniotic fluid was caused by the transplanted BMSCs, we detected their expression in the cultured BMSCs. The results of the cell immunofluorescence staining showed that cultured BMSCs in vitro could secrete these cytokines (Figure 3A). We also observed the expression of these factors in the BMSCs engrafted spinal cords. The expressions of Activin A, NGF, BDNF, CNTF, and CXCR4 were detected in both the BMSCs and neighboring host neural cells in the defective region of the spinal cord (Figure 3B). These results indicated that the transplanted BMSCs could secrete these factors by itself and also induces the secretion of neighboring host cells which might be conducive to improvement of the spinal cord microenvironment.

### 3.4 The upregulated Activin A, NGF, BDNF, CNTF, and CXCR4 in the amniotic fluid were responsible for the SBA repair

**FIGURE 4:**
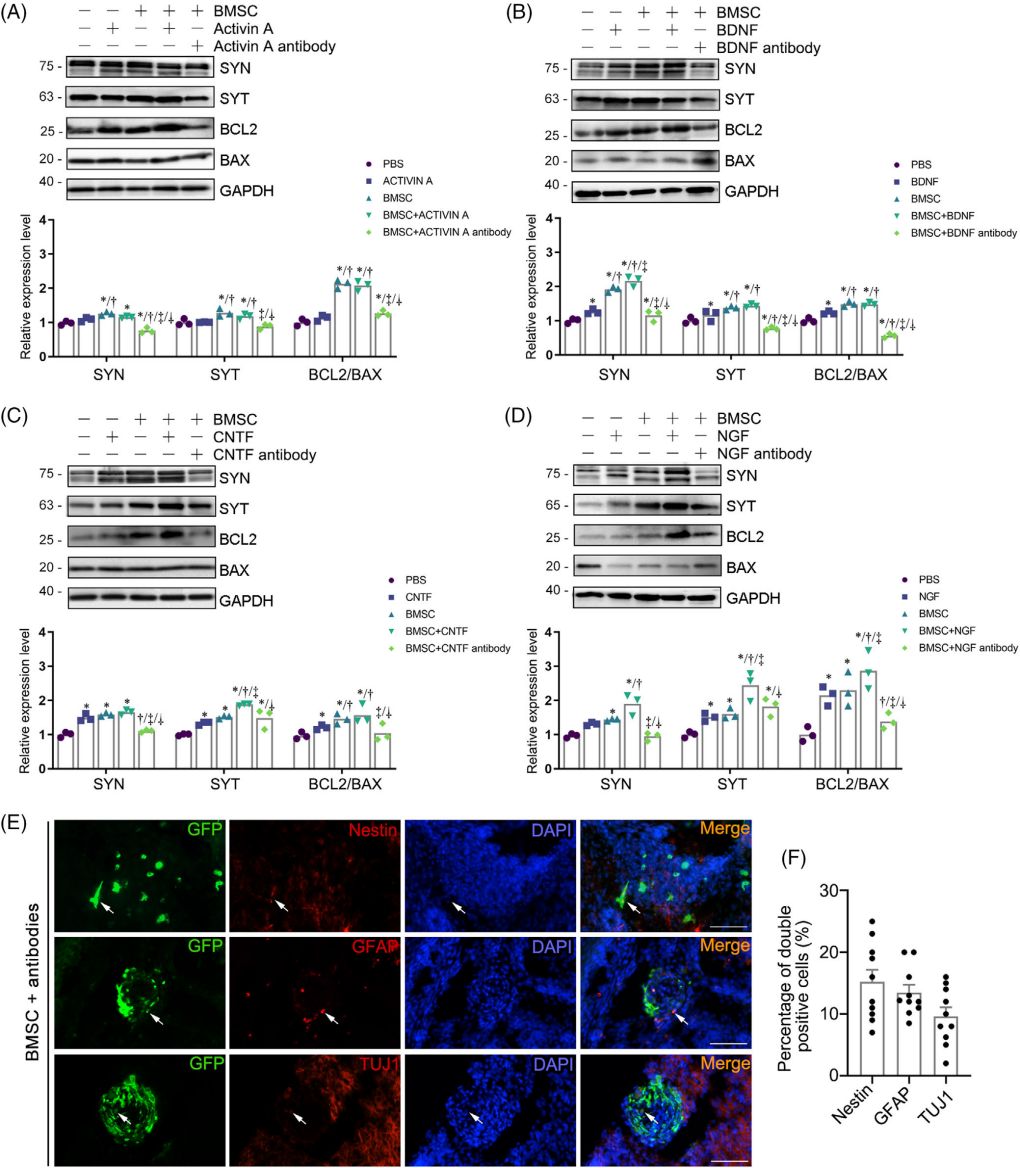
The anti‐apoptotic and pro‐differentiated effects after intervention by Activin A, BDNF, CNTF, and NGF or their inhibitors. (A‐D) Protein levels of SYN, SYT, BCL2/BAX in spinal cords from NTD fetuses after intra‐amniotic injection of PBS, Activin A/BDNF/CNTF/NGF, BMSCs, Activin A/BDNF/CNTF/NGF + BMSCs, or BMSCs + Activin A/BDNF/CNTF/NGF antibody. The column diagram shows the quantification of relative protein levels determined from immunoblots. *Significant difference compared to PBS‐injected group, ^†^Significant difference compared to Activin A/BDNF/CNTF/NGF‐injected group, ^‡^Significant difference compared to BMSC‐injected group, ^⸸^Significant difference compared to the BMSCs + Activin A/BDNF/CNTF/NGF group, *p* < 0.05. (E) Representative immunofluorescence staining images of the Nestin (red, top row), GFAP (red, middle row), and TUJ1 (red, bottom row) in the defective spinal cords after intra‐amniotic BMSCs + antibodies (including four antibodies against Activin A, BDNF, CNTF, and NGF) transplantation. The engrafted BMSCs were labeled with GFP (green), and the double positive cells are marked by the arrows. The nuclei were stained blue with DAPI. Scale bars: 50 μm. (F) Quantitative analysis of the Nestin, GFAP, and TUJ1 positive cells in the defective spinal cords with BMSCs engraftment after intra‐amniotic BMSCs + antibody transplantation. The percentage of BMSCs expressing these markers was determined as the number of double positive cells/total number of GFP positive cells (*n* = 10 sections).

To clarify whether the upregulated neurotrophic factors in the amniotic fluid and spinal cords are responsible for the observed amelioration of SBA, we evaluated the effects of the in utero intra‐amniotic injection of Activin A, NGF, BDNF, and CNTF with or without the BMSCs and their inhibitors in combination with the BMSCs on E15 SBA fetuses. The results of immunoblot analysis for apoptosis‐ and neurogenesis‐related proteins in the whole spinal column showed that compared to the PBS injection group, injection of BDNF, CNTF, and NGF without the BMSCs into the amniotic fluid could significantly upregulate the Bcl2/Bax ratio and the synaptic development‐related proteins SYN and SYT. Injection of Activin A only had little effect on the expression of BCL2/BAX, SYN, and SYT. Compared with the BMSC injection group, the protein expressions of SYN, SYT, and Bcl2/Bax were upregulated in the BDNF + BMSC, CNTF + BMSC and the NGF + BMSC injection groups. SYN protein expression was significantly upregulated in the BDNF + BMSC injection groups, and SYT was greatly upregulated in the CNTF + BMSC injection group. In addition, SYT and Bcl2/Bax were greatly upregulated in the NGF + BMSC injection group. These upregulation trends were inhibited by the intra‐amniotic inhibitors of these growth factors + BMSC injection. Taken altogether, our results indicate that the anti‐apoptotic and pro‐neurogenetic effects in the growth factor + BMSC groups were superior to that of the growth factor only or the BMSC only injection groups (Figure 4A–D). The immunofluorescence staining results confirmed that the inhibitors of Activin A, NGF, BDNF, and CNTF inhibited the expression of Nestin, GFAP, and TUJ1 in the transplanted BMSCs (Figure 4E). To better analyze the effect of growth and trophic factor antibodies treatment on the neural differentiation of the transplanted BMSCs, we observed the expressions of Nestin, GFAP, and TUJ1 in the spinal cords of the fetuses treated with BMSCs + four antibodies (including four antibodies against Activin A, BDNF, CNTF, and NGF) using immunofluorescence staining. The results showed that compared with the BMSC injection group, the percentage of BMSCs that expressed Nestin, GFAP, and TUJ1 were decreased in the BMSC + factor antibodies treatment (18.6 ± 6.6% vs. 15.2 ± 5.7%, 26.7 ± 10.2% vs. 13.5 ± 3.6%, and 20.2 ± 9.3% vs. 9.6 ± 4.2%, respectively). The above results demonstrate that BMSCs delivered into the amniotic fluid might improve the microenvironment of the amniotic fluid via secreting these neurotrophic factors, which is conducive to anti‐apoptosis and pro‐neurogenesis of the spinal cord. All these proteins are potential candidates responsible for BMSC‐mediated neuronal protection.

**FIGURE 5:**
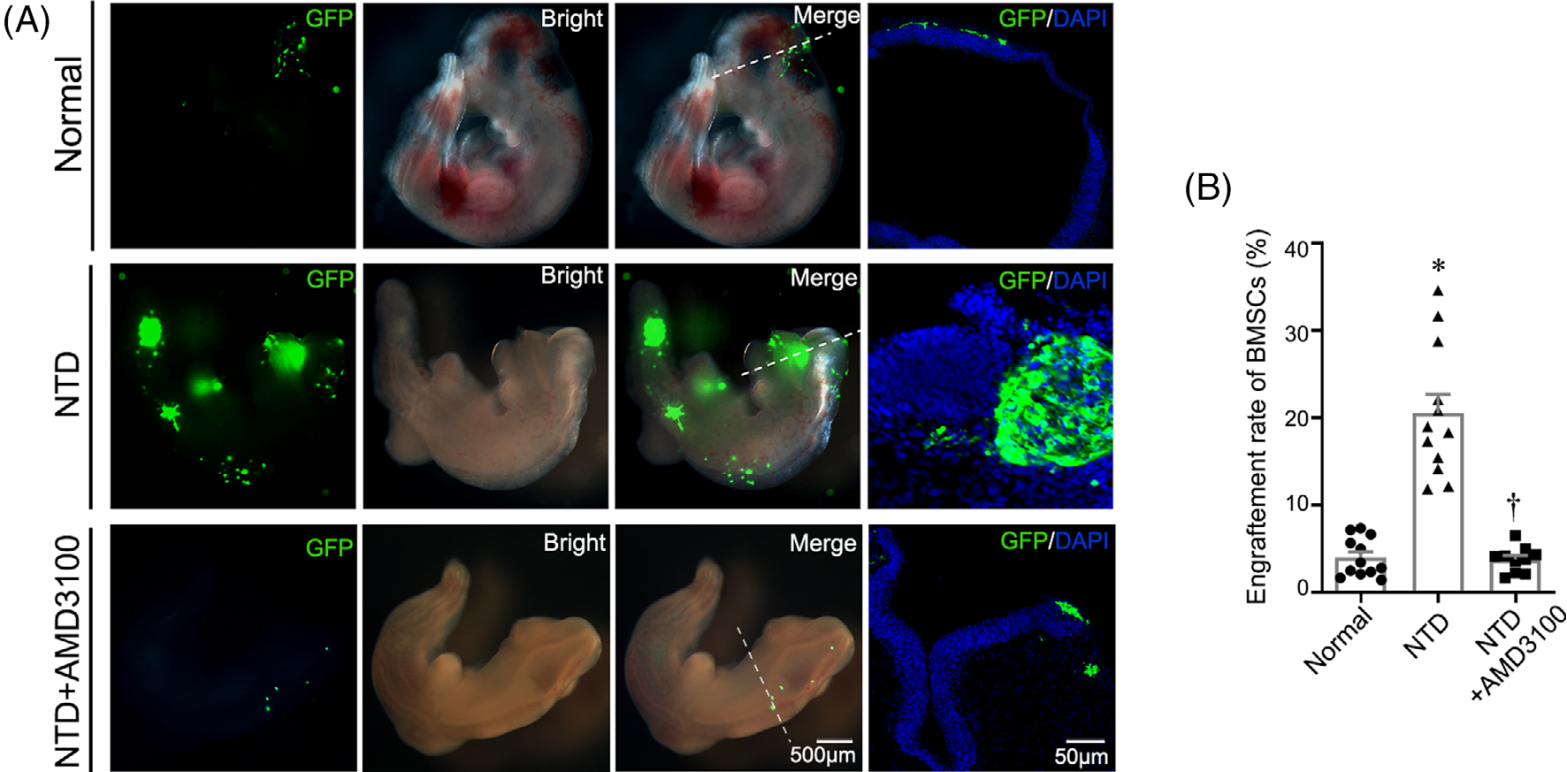
The lesion‐specific migration ability of BMSC was weakened after intervention with the CXCR4 inhibitor. (A) Representative fluorescent stereomicroscopic images of rat embryos after intra‐amniotic BMSC transplantation in a normal fetus, an NTD fetus, and an NTD fetus treated with AMD3100. The transverse sections were achieved along the white dotted lines. Scale bars: 500 μm (fluorescent stereomicroscopic images) and 50 μm (cryosection images). (B) Evaluation of the BMSC engraftment rate in normal fetuses (*n* = 12), NTD fetuses (*n* = 12), and NTD fetuses treated with AMD3100 (*n* = 10). The engraftment rates were determined by the total number of GFP +ve BMSCs in the continuous frozen sections/the number of transplanted BMSCs. *Significant difference compared with the normally developed embryos, ^†^Significant difference compared to the NTD fetuses, *p* < 0.05.

To verify whether the upregulated CXCR4 (the chemokine with the most obviously different expression after intra‐amniotic BMSC transplantation) in the amniotic fluid played a role in the lesion‐specific migration of BMSCs, we injected CXCR4 inhibitor + BMSCs into the amniotic fluid of ex vivo cultured NTD embryos. Our results show that the engraftment of GFP + ve BMSCs was significantly decreased in the CXCR4 inhibitor treated groups, which means that intra‐amniotic transplanted‐BMSCs can secrete CXCR4 to participate in BMSC‐directed migration (Figure 5A,B).

## 4 DISCUSSION

Currently, SBA represents the most prevalent congenital malformation of the nervous system, but the treatment options remain limited and far from satisfactory. Due to the absence of skin and musculoskeletal coverage, the exposure of unprotected spinal cord to neurotoxic amniotic fluid appears to be more closely associated with the final outcome of neurological function defects in SBA fetuses. For the first time, we demonstrate in this study that after delivery into the amniotic cavity, BMSCs secrete neurotrophic factors and chemokines to improve the amniotic fluid and neural tube microenvironments, inhibit neural apoptosis, and promote neural differentiation in the spinal cords of rat fetuses with SBA. Because the secondary injury of SBA is mainly caused by chemical stimulation of amniotic fluid components, improving the microenvironment of the amniotic fluid is of great importance for SBA treatment.

Previously, we reported that intra‐amniotic injected BMSCs can lead to a 29.94% reduction in the area of the skin lesion and a shorter latency and higher MEP amplitude in SBA fetuses via epithelial/neural regeneration. The therapeutic efficacy of BMSCs depends on their ability to multi‐differentiate.^33^ In the present study, we found that the therapeutic efficacy also depends on their paracrine effects, which lead to neurogenesis, anti‐apoptotic, and chemotaxis effects. Our study showed that besides the SBA‐lesion specific engraftment, many intra‐amniotic injected BMSCs also survived in the amniotic fluid. The paracrine signals of these BMSCs surviving in the amniotic fluid may be the main reason for the improvement in the amniotic fluid microenvironment. Studies have reported that permanent damage induced by the amniotic fluid is the primary cause of neurodegenerative injury in the exposed spinal cord of human fetus with SBA.^40^ Thus, improving the microenvironment of the amniotic fluid may prevent the secondary neural injury of SBA by reducing toxic stimulation of exposed neural tubes in the amniotic fluid.

In this research, we emphasized the secretion of cytokines after the intra‐amniotic BMSC injection in the amniotic fluid and spinal cord. Our study showed that the neurotrophic factors involved in the negative regulation of neuron apoptotic process, including NGF, CNTF, and BDNF, were significantly increased in the amniotic fluid after BMSC injection. These upregulated factors have been shown to play important roles in neural protection, neurite outgrowth, regulation of neuronal connections, and wound healing,^41^, ^42^ which might enhance the neuroprotective effect of BMSCs on the defective neural tube. Indeed, our intervention studies further demonstrated that intra‐amniotic BDNF, CNTF, or NGF injection could reduce apoptosis and promote neurogenetic and synaptic development‐related protein expression in fetuses with spinal bifida. Inhibiting the activity of these factors weakened the neuroprotective effect of BMSCs on defective spinal cords. During embryonic development, the survival and development of most neurons in the peripheral nervous system depend on trophic factors. BDNF and NGF belong to the neurotrophin gene family and play an important role in neuronal survival, neuronal differentiation, and maintenance of specific neuronal populations.^41^, ^43^ BDNF has been studied in detail with respect to its function on neuronal survival, formation, metabolism, and axonal growth.^41^, ^43^, ^44^ Our previous study indicated that intra‐amniotic adenovirally expressed BDNF injection can inhibit neural apoptosis and promote survival of sensory neurons in the rat fetuses with SBA.^35^ BDNF has also been shown to protect neurons and improve neurological function in models of spinal cord injury,^45^, ^46^ retinal injury,^44^, ^47^ and hearing diseases.^48^ NGF can directly repair injured nerves and regulate neuronal activity, neural connections, and synaptic plasticity.^45^ CNTF belongs to the neurotrophic cytokine family and can promote neurite outgrowth of neurons and astrocytes^49^ and reduce inflammation.^50^ CNTF can also regulate the survival and/or differentiation of many cell types, including neurons, adipocytes, oligodendrocytes, muscle cells, bone cells, and retinal cells; thus, it is widely used in treating neurodegenerative and metabolic diseases.^51^, ^52^ Local application of CNTF in the model of motoneuron diseases (such as amyotrophic lateral sclerosis) rescues loss of synaptic vesicles from the active zones of motor axon terminals to maintain the synaptic function,^53^ and systemic CNTF treatment in a progressive motor neuronopathy mice model could protect survival and inhibit axon loss.^54^ In the present study, the injection of only BDNF, CNTF, or NGF into the amniotic fluid could inhibit cell apoptosis and promote neurogenesis in the spinal cord of SBA fetuses. Inhibitors of BDNF, CNTF, and NGF reduced the anti‐apoptotic and pro‐differentiated effects of intra‐amniotic BMSC injection on defective spinal cords. These results suggest that BDNF, CNTF, and NGF participate in SBA repair through inhibiting apoptosis and promoting synaptogenesis. Activin A is a member of the transforming growth factor‐β superfamily^55^ and is involved in cellular growth, survival, differentiation, and remodeling, and also plays a crucial role during embryo and neural tube development.^56^, ^57^ Compared with NGF, BDNF, or CNTF injections, Activin A injections have a weaker effect of anti‐apoptosis and pro‐neurogenesis on defective spinal cords, although it has been reported that Activin A had effects on neuroprotection and neural stem cell fate decision during brain development.^58^ More reports suggest that Activin A is implicated in wound repair and fibrosis. Overexpression of Activin A could promote wound repair, skin morphogenesis, and scar formation.^59^ Our results demonstrated that intra‐amniotic Activin A injection caused no significant change in BCL2 / BAX, SYN, and SYT, which might be because Activin A mainly plays a role in skin repair. This study focused on apoptosis and neurogenesis, and we did not observe the effects on skin repair. Future research will focus on observing the effect of Activin A on skin repair.

Furthermore, we found that several soluble chemokines, including CXCR4, IP10, MCP1, Fractalkine, and IL1A, were markedly upregulated. It has been reported that the chemokine system is involved in neural development.^60^, ^61^, ^62^ Chemokines and their receptors are widely expressed in the nervous system where they regulate stem cell migration, axonal pathfinding, and neuroinflammatory responses.^60^, ^61^ CXCR4, the chemokine with the most obviously different expression after intra‐amniotic BMSC transplantation, has been reported to have an important effect on the migration of transplanted BMSCs after intra‐amniotic BMSC transplantation. Over‐expression of CXCR4 in MSCs by genetic modification could enhance MSC engraftment in injured tissue repair.^62^ Our study confirmed that inhibition of CXCR4 activity reduced the engraftment rate of BMSCs in SBA embryos. The upregulated chemokines in our study might be involved in lesion‐specific engraftment of the BMSCs, leading to effective tissue repair. We understand that BMSCs possess profound immunomodulatory effects, and several important inflammatory cytokines involved in inflammation related signaling pathways, including GMCSF, TLR4, IL2, IL4, IL10, and IL13, were significantly increased in the BMSCs transplanted amniotic fluid. GMCSF has some neuroprotective effects. These include promoting cell proliferation and differentiation, inhibiting the release of proinflammatory factors, and cell apoptosis.^63^ Elevated anti‐inflammatory cytokines, such as IL4 and IL13, are known to have a direct protective effect on cellular survival, proliferation, and neural regeneration, while increased IL10 is involved in cell survival and may promote recovery after spinal cord injury.^64^ These factors are involved in mediating immunomodulation and might contribute to the neuroprotective effects of the BMSCs.^63^, ^64^, ^65^, ^66^ In the future, more trials will be required to evaluate the effect of these increased inflammatory cytokines on neuroprotection in SBA fetuses.

In conclusion, results showed that intra‐amniotic BMSC injection improves the microenvironments of the amniotic fluid and spinal cord to prevent secondary damage in rat fetal spina bifida models, which might be an important mechanism of BMSC injection‐induced SBA recovery. Based on the paracrine characteristics of BMSCs, the mechanism of BMSC transplantation improves the microenvironment of the recipient lesion area, and this is primarily attributed to: (i) the secretion of neurotrophic factors and chemokines; (ii) immunomodulatory and anti‐inflammatory effects; (iii) the secretion of extracellular vesicles. In addition, it has been reported that the molecules secreted by MSCs perform an effective role as mediators which either directly activate the target cells or stimulate neighboring cells to secrete active factors.^67^ Our results showed that the transplanted BMSCs secreted a variety of important protein molecules that play irreplaceable roles by inhibiting cell apoptosis, promoting cell survival and synapses connections, and regulating the inflammatory response in BMSC‐transplanted amniotic fluid and the spinal cord, which might be critical for the survival and neuro‐differentiation of cells in the uncovered spinal tissue. Indeed, the intervention experiments showed that the growth and nutritional factors Activin A, NGF, BDNF, and CNTF were involved in SBA repair by regulating apoptosis and synaptic development, while chemokine CXCR4 was related to the lesion‐specific engraftment of BMSCs. The data provided in the present study indicate that intra‐amniotic BMSC together with cytokines injection might serve as a new strategy for prenatal spina bifida treatment. Combined with our previous research results, we found intra‐amniotic transplanted BMSC has strong potential to treat SBA by spontaneously migrating to the SBA lesion, multi‐differentiating into defective cells and improving the microenvironment to avoid further damage and promote SBA repair. Fetal SBA can be evaluated by ultrasonography from the 12th week of gestation, but the prenatal repair of patients with SBA can only be carried out between the 19th and 27th weeks of gestation; during this period, the neural damage caused by SBA may be irreversible. Ultrasound‐guided amniocentesis can be performed in early and middle pregnancy, and is less traumatic for both mothers and fetuses. It provides a convenient and feasible method for the application of intra‐amniotic stem cells or other reagent injections, which can be used to treat NTD immediately after ultrasound diagnosis. It can not only solve the problem of having a very short time window in fetal surgery, but can also solve issues with irreversible neural damage at the time of fetal surgery. Even though intra‐amniotic BMSC injection may not completely repair the multi‐tissue defects in NTD fetuses, it can be used as an important auxiliary treatment in fetal surgery. Clinical trials are needed to estimate the efficacy and safety of intra‐amniotic BMSC transplantation.

## AUTHOR CONTRIBUTIONS

Z.W.Y., X.W.W. and W.M. conceived the project. X.W.W. and W.M. designed and performed the experiments, analyzed the data, and drafted the manuscript and figures. H.G., D.L., W.T.L., S.Y.C., S.S.J., T.C.H., Y.W.H. W.L.W. and Y.Z.B. assisted in the preparation of the manuscript. Z.W.Y. supervised the study. All authors read and approved the final manuscript.

## CONFLICT OF INTEREST

The authors indicated no potential conflicts of interest.

## Data Availability

All data generated or analysed during this study are included in this published article and its supplementary files.
